# Social Cognition in Autism Spectrum Disorder Across the Adult Lifespan: Influence of Age and Sex on Reading the Mind in the Eyes Task in a Cross-sectional Sample

**DOI:** 10.3389/fnint.2020.571408

**Published:** 2020-09-04

**Authors:** Broc A. Pagni, Melissa J. M. Walsh, Carly Rogers, B. Blair Braden

**Affiliations:** Autism Brain Aging Laboratory, Arizona State University, College of Health Solutions, Tempe, AZ, United States

**Keywords:** autism, social cognition, aging, sex differences, adulthood, theory of mind, reading the mind in the eyes, multiple regression

## Abstract

**Background**: Approximately 50,000 U.S. teens with autism spectrum disorder (ASD) become adults every year, however little is known regarding how age influences social cognition and if men and women with ASD are differentially impacted across the adult lifespan. Social cognition declines non-linearly with age in neurotypical (NT) adults. Moreover, sex differences have been observed on RME tasks in NT adults but not adults with ASD, although aging effects have been largely ignored.

**Objective**: This cross-sectional study examined the influence of age and sex on social cognition in adults with ASD compared to NT adults.

**Methods**: The Reading the Mind in the Eyes (RME) task was administered to evaluate the theory of mind abilities in 95 adults with ASD and 82 NT adults ages 18–71 years. The main effects of diagnosis, age, and sex, as well as two-way and three-way interaction were modeled using linear and quadratic aging terms in a multiple regression analysis.

**Results**: A main effect of diagnosis was observed, indicating poorer performance in adults with ASD relative to NT adults. Age and sex interactions were nonsignificant.

**Discussion**: We replicated previous findings of reduced theory of mind (ToM) abilities in adults with ASD, compared to NT adults. While interactions were nonsignificant, visual inspection of quadratic age curves indicated the possibility of unique ToM trajectories in men and women with and without ASD that should be investigated in larger longitudinal studies.

## Introduction

Individuals with autism spectrum disorder (ASD) face marked challenges in social and communicative functioning, including difficulties in understanding other people’s goals, intentions, and emotional states, known as the theory of mind (ToM; Baron-Cohen et al., 2015). In the United States, approximately 50,000 teens with ASD age into adulthood every year (Shattuck et al., 2012). Further, there is a growing population of elderly with ASD, which is expected to reach 700,000 individuals over the age of 65 by 2030 (Piven and Rabins, 2011). Despite findings in neurotypical (NT) adults that growing old negatively impacts socio-communicative functioning, it is unclear how age influences these skills in this growing population of adult men and women with ASD (Carstensen et al., 2003; Pardini and Nichelli, 2009).

Deficits in social cognition have been reported in ASD across a variety of psychometrically-validated ToM tasks, including the Reading the Mind in the Eyes (RME) task which has been extensively examined in children and adolescents, and to lesser extent adults (Baron-Cohen et al., 2015; Peñuelas-Calvo et al., 2019; Baksh et al., 2020). The RME task measures mentalizing abilities—also referred to as mindreading—and correlates strongly with other ToM tasks including Strange Stories and Faux Pas, suggesting an overlap in the socio-cognitive constructs measured. Compared to NT adults, intellectually-able adults with ASD reliably demonstrate poorer performance on the RME, Mental State Voices, Strange Stories, and Faux Pas tests (Kleinman et al., 2001; Baron-Cohen and Wheelwright, 2004; Spek et al., 2010; Baker et al., 2014). RME scores in adults with ASD inversely correlate with autism symptom severity and positively correlate with IQ on reading the mind in the voice task in ASD (Rutherford et al., 2002; Golan et al., 2007). Together, these studies suggest pronounced social cognition difficulties related to ToM in ASD that persist into adulthood, potentially moderated by IQ.

Aging in healthy adults is associated with diminished social functioning and declines in ToM performance (Carstensen et al., 2003; Moran, 2013; El Haj et al., 2016). For example, NTs show age-related declines on RME scores, with the noticeable decline starting around the fifth decade of life (Pardini and Nichelli, 2009). Such age-related decline in ToM has been demonstrated across many task types, domains, and modalities, suggesting broad aging effects on social cognition (McKinnon and Moscovitch, 2007; Henry et al., 2013; Baksh et al., 2018) which may also be influenced by education and IQ (Miguel et al., 2017). Multiple groups have identified an inverted-U shape relationship between age and social cognition in cross-sectional NT samples, suggesting middle-aged adults may outperform young and old adults (Williams et al., 2009; O’Brien et al., 2013). Conversely, in a cross-sectional ASD study, middle-aged adults displayed greater autistic symptoms, including those related to social functioning, compared to younger and older adults with ASD (Lever and Geurts, 2018). While ToM studies in ASD have primarily focused on developmental trajectories of children and adolescents (Steele et al., 2003; Peterson and Slaughter, 2009; Scheeren et al., 2013), assessment across the adult lifespan has yet to be performed. For example, adolescents outperform children with ASD (Scheeren et al., 2013); however, we know very little about ToM abilities in young, middle, and elder adulthood. Given substantial evidence of ToM impairments in ASD and age-related decline in NTs, we suspect the age-related decline in adults with ASD may compound ToM difficulties.

Sex differences in socio-emotional processing begin in early development and persist into adulthood (Connellan et al., 2000; Knickmeyer and Baron-Cohen, 2006; Proverbio, 2017; Olderbak et al., 2019). For example, female children and adults score higher on empathy compared to male counterparts (Wheelwright et al., 2006; Auyeung et al., 2009). Studies measuring social personality differences between men and women point toward a genetic and neural basis, suggesting sex-specific biomarkers may aid in tracking social functioning across the lifespan (Kana and Travers, 2012; Pearce et al., 2019). Williams et al. (2009) found a three-way interaction between age, sex, and explicit emotion recognition in a large cross-sectional sample, showing women have greater accuracy for identifying sadness and fear in older decades while men have greater accuracy for identifying anger in younger decades. Specific to RME, a meta-analysis in NT adults found females perform better than males, although effect sizes were estimated to be small and aging influences were not examined (Kirkland et al., 2013).

Biological and behavioral differences between men and women with ASD suggest careful consideration is needed to achieve an unbiased diagnosis and tailored treatment plans across the ASD lifespan (Baron-Cohen et al., 2005; Carter, 2007; Holtmann et al., 2007; Lai et al., 2011; Begeer et al., 2013; Zeestraten et al., 2017). For example, females on average are diagnosed later than their male counterparts and toddler girls present greater communication deficits while boys greater restricted and repetitive behaviors (Carter et al., 2007; Hartley and Sikora, 2009). However, social symptom profiles may change as a function of age, as adult women with ASD report fewer socio-communication difficulties than men despite both sexes showing similar degrees of empathy (Baron-Cohen and Wheelwright, 2004; Auyeung et al., 2009; Lai et al., 2011). In a well-powered, unbiased sample of adult men and women with ASD, Baron-Cohen et al. (2015) found no evidence of sex differences on RME performance. However, there is still ongoing debate regarding the pervasiveness of social symptoms and a need for mapping developmental trajectories across adulthood (Grove et al., 2017; Schuck et al., 2019). Importantly, the investigation into potential idiosyncratic aging trajectories between the sexes is necessary to ensure sex differences are not obfuscated when collapsing across age.

This study is the first to examine age-related influences on RME performance across a broad age range of adults with and without ASD. We conducted a multiple regression analysis to identify 2-way and 3-way interactions between diagnosis, age, and sex and considered linear and quadratic relationships. Further, we attempted to confirm previous findings of impaired ToM performance in ASD, sex differences in healthy controls but not ASD, and age-related decline in NTs. We hypothesized: (1) a main effect of diagnosis with adults with ASD performing worse than healthy controls; (2) sex by diagnosis interaction showing superior female performance in NTs but not ASD; and (3) a diagnosis by age by sex interaction showing unique aging trajectories for women and men, dependent on diagnosis. Finally, based on previous research, we anticipated quadratic age effects would best capture the relationship between age and ToM performance.

## Materials and Methods

### Participants

Data in this study were collected from adults with ASD (*n* = 95) and NT adults (*n* = 82) aged 18–71 years similar in age, IQ, and sex distribution (Table 1). Participants were recruited *via* local advertisement and word of mouth through community organizations. Participants with ASD were mainly recruited from the Southwest Autism Research and Resource Center (SARRC). All participants with ASD met DSM-V criteria for ASD diagnosis and were confirmed by a research-reliable rater who administered the Autism Diagnostic Observation Schedule-2, module 4 (ADOS-2, Lord et al., 2012). NT participants had neither a previous ASD diagnosis nor a history of a first-degree relative with ASD. NT participants were screened using the Social Responsiveness Scale (SRS-2; Constantino and Gruber, 2005) in which negative results were necessary for inclusion in this study. IQ estimates were provided by the Kaufman Brief Intelligence Test 2^nd^ edition (KBIT-2, Kaufman and Kaufman, 1990). To rule out cognitive disorders, a minimum of 26 was required on the Mini-Mental State Examination (MMSE, Cockrell and Folstein, 1988). Exclusion criteria for both groups included: a history of a traumatic brain injury (qualified as resulting in loss of consciousness), substance use disorder, schizophrenia, and seizure disorders. A history of depression or anxiety were not used as exclusionary criteria considering high rates of comorbidity in ASD (Wigham et al., 2017). Informed consent was obtained from all participants and the study was approved by the Institutional Review boards for both Barrow Neurological Institute and SARRC. All work-related to this study aligned with the ethical standards as outlined in the Declaration of Helsinki (2000).

**Table 1:** Diagnostic groups demographics and statistics.

	NT (*n* = 82)	ASD (*n* = 95)	Statistics
Age (years)	Mean = 41.67 ± 16.794 Range = 18–70	Mean = 39.93 ± 16.469 Range = 18–71	*t*_(175)_ = 0.696 *p* = 0.49
Sex (males/females)	48/34	67/28	$\chi_{\left(175\right)}^{2}$ = 2.780 *p* = 0.10
KBIT-2^a^ composite	Mean = 108.56 ± 11.551 Range = 85–141	Mean = 106.64 ± 14.543 Range = 70–139	*t*_(173.849)_ = 0.978 *p* = 0.33

*^a^Kaufman Brief Intelligence Test-2*.

### RME Task

Social cognition abilities were assessed using the RME ToM task. In the RME, participants were shown 36 different pictures depicting human eyes with a small portion of the face directly surrounding the eyes. Participants were instructed to select the emotional state that best represented what the person is feeling from four available options. Participants had access to definitions of the words to refer to if they were unsure of the meaning. This mind-reading paradigm is designed to test ToM abilities which required participants to draw inferences based on limited facial expression cues (Ekman and Friesen, 1978) given sufficient information for reasonable accuracy (Kleinke, 1986). The RME assessment is scored based on the number of correctly identified emotional states out of 36. All pictures and stimuli presented were of equal size and in black and white.

### Statistical Analyses

Independent samples *t*-tests or chi-squared tests were conducted to ensure ASD and NT groups were similar in respect to age, sex distribution, and IQ. A multiple regression analysis was performed with the following factors: diagnosis, age, age squared, sex, diagnosis by age, diagnosis by age-squared, diagnosis by sex, age by sex, age-squared by sex, diagnosis by age by sex, and diagnosis by age-squared by sex, with IQ as a covariate. Alpha was set at *p* < 0.05 using the Statistical Package of Social Sciences (SPSS).

## Results

**Figure 1:**
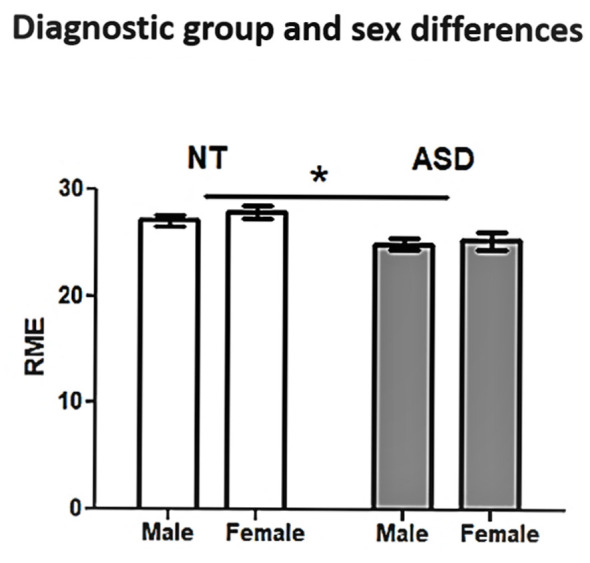
Reading the Mind in the Eyes (RME) performance by diagnosis group and sex. Neurotypical (NT) adults performed better than adults with autism spectrum disorder (ASD). *p < 0.05.

**Figure 2:**
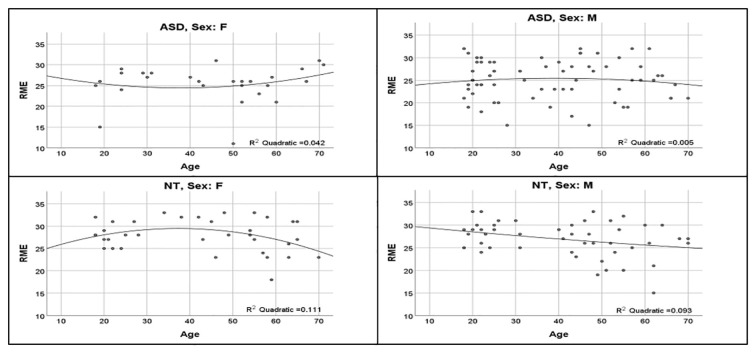
Influence of age across diagnosis group and sex. Non-significant quadratic age effects show upright U-shaped curve for women with ASD, inverted-U shape for NT women and men with ASD, and downward linear slope for NT men.

ASD and NT groups were similar with respect to age (*t*_(175)_ = 0.70, *p* = 0.49), sex distribution ($\chi_{\left(175\right)}^{2}$ = 2.78, *p* = 0.10), and IQ (*t*_(173.85)_ = 0.98, *p* = 0.33; Table 1). The regression model was significant (*F*_(12,164)_ = 4.324, *p* < 0.0001), revealing a significant main effect for diagnosis (*t* = −2.95, *p* = 0.004; Figure 1; Table 2). Overall, NT adults performed better on RME compared to adults with ASD. The main effect of sex was nonsignificant (*t* = −1.79, *p* = 0.075; Table 2). Additionally, two-way diagnosis by sex (*t* = 1.83, *p* = 0.068; Figure 1; Table 2) and three-way quadratic age by diagnosis by sex interactions (*t* = −1.79, *p* = 0.075; Figure 2; Table 2) were nonsignificant. No other main effects or interactions were detected.

**Table 2:** Reading the Mind in the Eyes (RME) multiple regression of diagnosis, age, and sex main effects and interactions.

Model: *F*_(12,164)_ = 4.324, *p* < 0.0001; Adjusted *R*^2^ = 0.185	Beta coefficient	*t*-statistic	*p*-value	CI
Diagnosis	−4.686	−2.946	0.004*	−7.83 to −1.55
Age	−0.048	−1.237	0.218	−0.12–0.03
Age-squared	−0.004	−1.343	0.181	−0.01–0.002
Sex	−2.549	−1.791	0.075	−5.36–0.26
Diagnosis × Age	0.019	0.315	0.753	−0.10–0.14
Diagnosis × Age-squared	0.007	1.693	0.092	−0.001–0.16
Diagnosis × Sex	3.583	1.834	0.068	−0.27–7.44
Age × Sex	−0.025	−0.492	0.623	−0.13–0.08
Age-squared × Sex	0.005	1.161	0.247	−0.003–0.01
Diagnosis × Age × Sex	0.026	0.346	0.730	−0.12–0.18
Diagnosis × Age-squared × Sex	0.010	−1.790	0.075	−0.02–0.001
Kaufman Brief Intelligence Test—Comprehensive Score	0.113	6.004	<0.0001	0.07–0.16

*p < 0.05.

## Discussion

In a cross-sectional sample featuring a wide adult age range, we compared ToM abilities in adults with ASD to NT adults by examining the influence of: (1) diagnosis; (2) age; (3) sex; and (4) two-way and three-way interactions between these factors; age was modeled *via* linear and quadratic terms to capture specific aging trajectories across the adult lifespan. Our results confirm adults with ASD perform worse on RME than NT adults; women and men with ASD perform equivalently, and NT women performed slightly better than NT men. Most novel to this study, visual inspection of the non-significant 3-way interaction provided a preliminary indication of unique quadratic aging profiles for adults with ASD and NT adults that may be sex-dependent.

This study is the first to examine age-related influences on RME performance across a broad age range of adults with and without ASD. Previous research corroborates age-associated differences on ToM tasks in other populations, however, aging effects in adults with ASD have yet to be systematically examined using the RME task. Although the 3-way interaction was nonsignificant, visual inspection of aging trajectories hints at the possibility of different quadratic relationships between RME performance and age for individuals belonging to specific diagnostic/sex categories. Albeit highly speculative, the qualitative patterns observed can be described as: (i) NT women showing an inverted-U; (ii) NT men a linear decline; (iii) women with ASD a U-shaped pattern; and (iv) men with ASD a flattened inverted-U (Figure 2). Higher-order interactions require exceptionally high power even with large samples, which may explain why this interaction was nonsignificant. For example, the sample size required to detect a three-way interaction is fourfold that required for a two-way interaction of the same effect size, warranting a discussion of the present quadratic aging interaction (Heo and Leon, 2010; Henry et al., 2018). Our regression model shows NT women and men perform similarly around 18–25 years of age; women and men with ASD also perform similarly at this age range, although worse than NTs. NT females show an inverted-U shape, with improvements in performance occurring up until middle-age before declining whereas NT men show a linear decline over time, in line with age-related declines in social cognition reported in NT adults (Pardini and Nichelli, 2009; Castelli et al., 2010; Moran, 2013; El Haj et al., 2016). Interestingly, men with ASD show a similar inverted-U shape to NT women, however, their curve is shifted downward and flattened whereas women with ASD show a U-shape, with RME scores at the lowest during middle-age. The aging curves we observed in women with ASD align with Lever and Geurts (2018), indicating greater social-related ASD difficulties at middle-age. However, their study did not investigate sex differences in trajectories; our study may suggest that quadratic symptom trajectories are driven by female participants. Moreover, these findings converge with notions of altered aging trajectories in adults with ASD relative to NT adults (Lever and Geurts, 2018).

Importantly, there is evidence from the general psychopathology literature suggesting females experience unique symptom aging trajectories, influenced by the interaction between environmental stressors and sex hormones. Furthermore, menopause remains an understudied stage in lifespan development in ASD (Moseley et al., 2020), which may play a role in symptom and cognitive dysregulation. Converging evidence suggests atypical sex-specific longitudinal development in ASD may be driven by atypical functional connectivity in the sensorimotor system, default mode network, salience network, and central executive network (Ypma et al., 2016; Hodes and Epperson, 2019; Lawrence et al., 2019, 2020; Kozhemiako et al., 2020). Future research should examine these systems to determine if they underlie sex-specific effects in social cognition across the adult ASD lifespan.

These disparate aging trajectories may provide insight into the underlying neural circuitry subserving ToM abilities and inform future therapies to preserve ToM functioning throughout the adult lifespan. Neuroimaging studies have identified idiosyncratic patterns of left inferior frontal gyrus (IFG) activity, a node in the executive network, across different age groups during RME. For example, Moor et al. (2012) found greater left IFG activity in young adolescents (10–12-year-olds) relative to young adults (19–23-year-olds) while Castelli et al. (2010) found greater left IFG activity in older adults (mean age = 65.2) relative to younger adults (mean age = 25.2) during mindreading. Future studies should interrogate the neural substrates of ToM to better understand age-related changes in social cognition across the adult ASD lifespan.

We also replicated several previous findings of diagnosis differences on the RME task. Our diagnosis main effect indicated adults with ASD performed worse compared to NT adults, in line with other RME studies in adults with ASD (Rutherford et al., 2002; Holt et al., 2014; Baron-Cohen et al., 2015). Better social cognitive abilities have been demonstrated in NT adults using other ToM tasks including Mental State Voices, Strange Stories, and Faux Pas tests, which correlate with diminished empathy in intellectually-abled adults with ASD (Kleinman et al., 2001; Baron-Cohen and Wheelwright, 2004; Spek et al., 2010; Baker et al., 2014). Together these studies reflect wide-sweeping social cognition impairments in adults with ASD.

Although the main effect of sex and diagnosis by sex interaction was nonsignificant, on average women performed better than men on the RME tasks. This relationship appears to be driven by NT women outperforming NT men, consistent with other reports suggesting a female advantage in NT adults, but not adults with ASD (Baron-Cohen et al., 2006, 2015; Hall et al., 2010; Kirkland et al., 2013; Olderbak et al., 2019). Corroborating this, an RME meta-analysis estimated small female advantage effects on RME performances, which likely explains why our interaction was nonsignificant rather than significant (Kirkland et al., 2013). This female advantage has been demonstrated using a diverse array of ToM tests including RME, Faux Pas, and false belief (Baron-Cohen et al., 1997, 2015; Hall et al., 2010; Koch and Tononi, 2011), although others have suggested sex differences may be dissociated using cognitive- and affective-specific ToM tasks since a male advantage has been shown on cognitive ToM tasks such as the “Cartoon” task (Russell et al., 2007). Such discrepancies may reflect nuanced NT sex differences in facial scanning, cognitive and affective processing, and brain function underlying ToM abilities (Hall et al., 2010). For example, women fixate on the eyes longer and more frequently than men—both of which correlate with greater accuracy and speed in facial recognition. Sex differences are also apparent neurobiologically, as NT men show greater activation in the left IFG during RME compared to NT women (Hall et al., 2010). Altered activation patterns in the IFG across the lifespan between women and men should be further examined to delineate relationships between social cognition, sex, and age. As suggested by Russell et al. (2007), NT men may rely more heavily on cognitive systemizing strategies whereas NT women rely more heavily on affective processing by utilizing features of the eyes more to decode emotional states, lending them to perform better on the affective RME ToM task.

The absence of sex differences in our sample of adults with ASD, when collapsed across ages, is consistent with reports from Baron-Cohen et al. (2015) who showed no differences between men and women on RME in a large, non-male biased sample of adults with ASD (*n*~400). These findings support the “Extreme Male Brain” theory, which postulates the ASD neuro-cognitive-phenotype is hyper-masculinized (Baron-Cohen et al., 2005; Knickmeyer and Baron-Cohen, 2006; Jung et al., 2015; Ypma et al., 2016), although neuroimaging studies in ASD point to different neural signatures between the sexes during ToM (Holt et al., 2014; Ypma et al., 2016). If indeed no sex differences exist, we might expect women with ASD to utilize strategies similar to men or for alternative cognitive and affective strategies to produce similar performance scores. The quadratic aging trajectories we observed in this wide-age range adult sample highlight how critical age may be to assessing neurobiological and behavioral sex differences in ASD. Further, ToM abilities should be probed using a variety of tasks, taking into account cognitive and affective ToM and attentional biases to facial cues.

### Limitations

Results are limited due to the cross-sectional nature of this study, the relatively modest sample size for three-way interactions, and the singular assessment of ToM. We used a cross-sectional sample to infer aging trajectories, whereas longitudinal analysis would be the gold standard for identifying differential aging trajectories. Further, our adult ASD sample of *n* = 95 was significantly smaller than Baron-Cohen’s (*n*~400). Therefore, a larger sample size may be needed to detect significant 2-way and 3-way interactions. Speculation has been made that the RME task may not be as sensitive to ToM capacities compared to newer instruments such as the Edinburgh Social Cognition Test (Baksh et al., 2018). While RME has been shown to effectively predict ToM, static facial stimuli used in the RME task do not capture dynamically changing socio-communicative facial gestures often used to infer the mental states of others. Additionally, an analysis of the RME stimulus set suggested valence, sex, and age may confound the instrument (Kynast and Schroeter, 2018). Future studies should utilize larger sample sizes and employ a battery of tasks indexing social cognition to elucidate the extent of ToM deficits. Lastly, differences in RME strategies between diagnostic groups, sexes, and age groups may conflate our findings and should be considered. Future research will benefit from the incorporation of eye-tracking and neuroimaging techniques to elucidate mechanisms underlying differences in ToM abilities.

## Conclusion

We replicated ASD-related impairments on a ToM task compared to an age- and IQ-matched NT sample, as well as provided some evidence for an absence of sex differences in ASD. Although differences in aging trajectories for adult men and women with and without ASD were nonsignificant, visual inspection of curves suggests there may be distinct aging patterns between men and women with and without ASD that warrant further investigation in an adequately powered longitudinal design. A nuanced characterization of ToM aging trajectories is still needed to inform personalized interventions seeking to improve quality of life and optimal social functioning across the adult lifespan.

## Data Availability Statement

The raw data supporting the conclusions of this article will be made available by the authors, without undue reservation.

## Ethics Statement

The studies involving human participants were reviewed and approved by Institutional Review Board, Arizona State University, STUDY00006088. The patients/participants provided their written informed consent to participate in this study.

## Author Contributions

BB: conceptualization, visualization, supervision, and funding acquisition. BP, MW, and BB: methodology, investigation and writing—review and editing. BP and BB: project administration and writing—original draft. BP, MW, CR, and BB: formal analysis.

## Conflict of Interest

The authors declare that the research was conducted in the absence of any commercial or financial relationships that could be construed as a potential conflict of interest.
